# Re-challenge of immune checkpoint inhibitor pembrolizumab with concurrent tocilizumab after prior grade 3 pneumonitis

**DOI:** 10.3332/ecancer.2023.1644

**Published:** 2023-12-08

**Authors:** Chitrakshi Nagpal, Sameer Rastogi, Shamim A Shamim, Sneha Prakash

**Affiliations:** 1 Department of Medicine, All India Institute of Medical Sciences, New Delhi 110029, India; 2 Department of Medical Oncology, Dr. B.R.A. Institute Rotary Cancer Hospital, All India Institute of Medical Sciences, New Delhi 110029, India; 3 Department of Nuclear Medicine, All India Institute of Medical Sciences, New Delhi 110029, India

**Keywords:** immune checkpoint inhibitors (ICI), pembrolizumab, immune-related adverse event (irAE), pneumonitis, re-challenge, tocilizumab

## Abstract

Immune checkpoint inhibitors (ICIs) are associated with specific immune-related adverse events (irAEs) which are unique compared to cytotoxic chemotherapy. For life-threatening adverse events including grade 3 or more, permanent discontinuation of the ICIs is recommended, albeit without much robust evidence. Safe re-challenge of ICIs with concurrent immunosuppression has been reported with irAEs like gastrointestinal toxicity and arthritis. Here we present a case of a lady with undifferentiated pleomorphic sarcoma with programmed death ligand1 expression, who showed a complete response to pembrolizumab used as third-line therapy. However, it had to be stopped after 22 doses when the patient developed grade 3 pneumonitis. In view of progression off pembrolizumab, and lack of other effective alternatives, pembrolizumab was re-challenged with concurrent interleukin-6 (IL-6) blockade using tocilizumab. This was based on preliminary evidence on the role of IL-6 in mediating the irAEs, especially pneumonitis. The patient re-attained a complete response with pembrolizumab. There was no recurrence of the pneumonitis after rechallenging, and there was partial radiographic resolution of the ICI-interstitial lung disease after the combination therapy.

## Introduction

Cytotoxic chemotherapy has been conventionally used for the treatment of metastatic soft tissue sarcoma (STS), with significant toxicity as well as dismal outcomes with a median overall survival of less than 1 year [1]. In the last few years, targeted therapy has been added to the armamentarium for STS. Pazopanib was the first approved targeted therapy for STS, which had a clinically significant 3 month improvement in the median progression-free survival (PFS) post one to four lines of chemotherapy [2]. Following this there was a steep rise in the approval of other targeted therapies like trabectedin and eribulin, however, responses with these agents were short-lived and did not culminate into any OS benefit.

Immune checkpoint inhibitors (ICI) have shown significant benefits in multiple cancers like melanomas, non-small cell lung cancer, renal cell carcinoma, urothelial malignancy, Hodgkin’ lymphoma, etc. Sarcomas are generally considered to be non-immunogenic tumours. SARC 028 was the first phase 2 trial that demonstrated the clinical activity of immunotherapy in two types of STS, undifferentiated pleomorphic sarcoma (UPS), and liposarcomas. Patients with UPS had an objective response rate of 40% and a 12-week PFS of 70%, and these responses were sustained with a median duration of 30 weeks [3].

Immunotherapy is associated with a different set of adverse effects compared to conventional chemotherapy. These are caused due to uncontrolled activation of the immune system [4]. These immune related adverse events (irAEs) commonly involve the gut (colitis), lung (pneumonitis), liver (hepatitis), skin (bullous skin disease, vitiligo) and endocrine organs (hypophysitis, thyroiditis). These are graded (grade 0 to grade 5) according to the National Cancer Institute Common Terminology Criteria for Adverse Events [5] based on severity. On the molecular level, these irAEs are due to ICI-mediated enhanced T cell activity against cross antigens, i.e., those shared by tumour and normal tissues may result in these irAEs [6].

Pneumonitis is an uncommon, but potentially serious toxicity associated with ICIs. Most of these cases are mild and resolve with drug discontinuation. Patients who develop grade 3 or 4 toxicity require immunosuppression and possible hospitalisation. Currently, it is recommended by both the American Society of Clinical Oncology (ASCO) and the European Society for Medical Oncology (ESMO) to permanently discontinue these drugs if there is a history of grade 3 or 4 irAEs, since it is well established that the risk of recurrence of toxicity is much higher in severe cases [7–9]. The rates of discontinuation of ICIs due to irAEs is very high, varying between 4% and 45% [10]. However, if all effective therapeutic options have been exhausted, one of the strategies to re-challenge ICIs while reducing the risk of recurrence of irAEs includes the use of concurrent immunosuppression [10].

Interleukin-6 (IL-6) is hypothesised to be associated with the pathogenesis of irAEs, including ICI-related interstitial lung disease (ILD) [4, 6, 11]. Apart from this, IL-6 blockade is also deemed to have anti-tumour effects [10]. Tocilizumab is an IL-6 receptor blocker, approved for auto-inflammatory diseases and cytokine release syndrome [4]. This case report highlights the concurrent use tocilizumab for the reintroduction of pembrolizumab in a patient of metastatic UPS with grade 3 pneumonitis to prevent the recurrence of ICI-ILD.

## Case presentation

A 63-year-old woman presented in September 2017 with a gradually progressive, painless swelling in the posterior aspect of the right knee for over 1 year. The patient underwent a wide local excision (WLE) with reconstruction at a local hospital. Histopathology of the resected specimen was suggestive of a spindle cell malignant tumour, likely UPS. Within 4 months of the surgery, the patient presented to us with a recurrence of the swelling. Whole body 18-fluorodeoxyglucose positron emission tomography (FDG PET) subsequently done which revealed recurrence just underneath the excision scar along with multiple enlarged FDG avid iliac and inguinal lymph nodes, and a left lung nodule. The patient underwent a repeat WLE with lymph node dissection, and microscopic examination of the tumour along with immunohistochemistry (IHC) was suggestive of a high-grade pleomorphic sarcoma, with nodal and distant lung metastasis. The patient was planned for doxorubicin-based chemotherapy. The patient was started on single agent doxorubicin in March 2018, however, there was progression after four cycles. The patient was then switched to a three-weekly gemcitabine with docetaxel regime in August 2018, which was discontinued after eight cycles in view of grade 4 anaemia. The patient progressed after a treatment-free interval of 3 months in the form of increasing size and number of pulmonary nodules. Since our patient had failed on two lines of chemotherapy, the post-operative specimen received after the second WLE was reviewed for programmed death ligand 1 (PD-L1) expression to consider using immunotherapy as the last resort. IHC using Ventana PD-L1 assay (SP263) [12] revealed a 25% PD-L1 expression. Since the patient had significantly distressing symptoms, we started a combination of three-weekly pembrolizumab (200 mg/dose) along with vascular endothelial growth factor receptor tyrosine kinase inhibitor pazopanib (800 mg daily) from May 2019 to help with rapid relief from symptoms [13].

The patient developed grade 2 infusion reaction, hypothyroidism and hypertension with pembrolizumab, which were easily managed with steroid pre-medication, levothyroxine supplementation and anti-hypertensives respectively. With only three cycles of this combination, there was a marked reduction in the size and avidity of the primary and metastatic lesions. The patient received a total of 22 cycles uneventfully and had a complete metabolic response (CMR) on response evaluation.

After receiving 22 cycles, however, the patient developed an acutely worsening dry cough, progressive dyspnoea on exertion (modified Medical Research Council grade 3) [14]. It was associated with desaturation at rest (room air saturation of 82%), and oxygen requirement by face mask at a maximum flow of 8 L/minute. There was no expectoration or fever at this time. A PET scan was performed at this time with the suspicion of progressive disease (PD), however, it revealed extensive mildly FDG avid areas of diffuse ground glassing and honeycombing in bilateral lung parenchyma. The primary lesion in the knee however had completely disappeared. A high-resolution computed tomography (HRCT) scan of the chest was done to characterise the lung lesions given this discordance between the primary and metastatic site. There were bilateral diffusely scattered subpleural and peribronchial patchy consolidations with surrounding ground-glass opacities in all the lobes, with some honeycombing in the upper lobe, suggestive of non-specific interstitial pneumonia (NSIP) pattern of ILD.

A pulmonary function testing (PFT), bronchoscopy with bronchoalveolar lavage (BAL) or trans-bronchial lung biopsy (TBLB) could not be done at this time because of the poor general condition. COVID-19 real time polymerase chain reaction (RT-PCR), rapid antigen test (RAT) as well as cartridge based nucleic acid amplification testing (CBNAAT) were done, which were negative. Routine blood counts were not suggestive of any leucocytosis. After ruling out infectious causes with as many non-invasive tests as possible, the patient was diagnosed as grade 3 ICI-ILD, NSIP pattern. Pembrolizumab was withheld keeping with the guidelines. The patient was started on prednisolone at 30 mg/day (0.5 mg/kg/day). A lower dose was given initially because infectious pathologies could not be completely ruled out with the limited testing. Later however, her prednisolone dose was increased, and mycophenolate mofetil (MMF) was started at 500 mg twice a day after no response to a higher dose of prednisolone for 48 hours. Gradually her supplemental oxygen, as well as steroids and MMF, were tapered off over a course of 6 months. PFT was done after 3 months of treatment once the patient was stabilised, and the patient had a forced vital capacity (FVC) of 0.82 L (42% predicted) with a normal ratio of forced expiratory volume in one second to the forced vital capacity (FEV1/FVC ratio) and diffusion capacity of lungs for carbon monoxide of 7.02 mmol (20% predicted), which were suggestive of persistent severe restriction of lung function.

**Figure 1.**
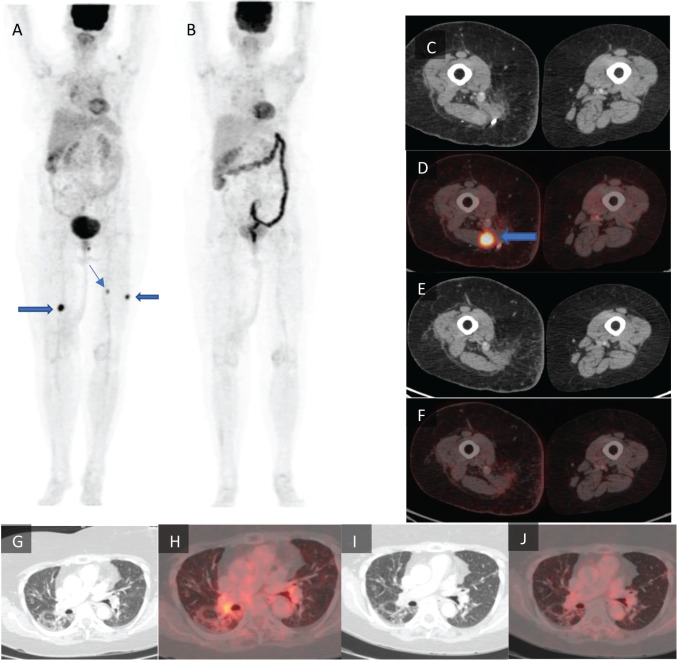
Maximum intensity projection images of (a): the pre-treatment PET-CT scan and (b): post-treatment PET-CT scan with the lower limb tumour lesions denoted by arrows. (c): Axial CT and (d): axial fused PET-CT of the pre-treatment scan with a lower limb tumour lesion denoted by arrow which is not seen in (e): the post-treatment axial CT and (f): axial fused PET-CT images of the same section. (g): Axial CT and (h): axial fused PET-CT images of the pre-treatment scan of the lungs in lung window. (i): Axial CT and (j): axial fused PET-CT of the same section of lungs in lung window.

**Figure 2.**
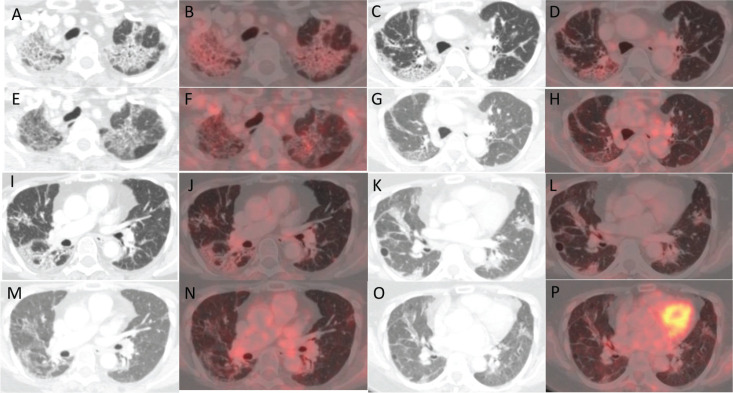
(a, c, i and k): Axial CT images of four different sections of lung showing changes with increased metabolic activity in corresponding (b, d, j and l): axial fused PET-CT images in the PET-CT scan after treatment with pembrolizumab. (e, g, m and O): Axial CT images of the same four sections of lung showing a reduction in the previously noted changes with reduced metabolic activity in corresponding (f, h, n and p): axial fused PET-CT images in the PET-CT scan done post treatment with pembrolizumab and tocilizumab.

Subsequently she was maintained on single agent pazopanib with close follow-up. After a pembrolizumab-free interval of 10 months, imaging revealed new FDG avid lesions in the muscles around the left shoulder, right semimembranosus, left vastus lateralis and left semitendinosus muscle, suggestive of PD. Since we were out of any effective alternatives, a decision to rechallenge pembrolizumab was made with concurrent tocilizumab, despite grade 3 ICI-ILD. The patient was given 200 mg pembrolizumab with 200 mg tocilizumab (4 mg/kg/dose) intravenously via infusion every 3 weeks. The patient tolerated this combination well with no recurrence of respiratory symptoms. PET scan done after three cycles revealed a CMR, with no progression of the ILD-related changes as shown in Figure 1. Currently, she has completed 13 cycles of this combination therapy and is in good general condition, with persistent CMR, as well as a partial resolution of ICI-ILD on imaging, as shown in Figure 2.

## Discussion

Sarcomas are considered to be immunologically cold tumours; hence conventional chemotherapy still remains the standard of care for advanced sarcomas. The SARC028 trial revealed clinically meaningful and sustained responses with immunotherapy, most of the responders being PD-L1 positive [3]. Our patient similarly had a 25% PDL1 expression and a very good response to immunotherapy, and also one of the major factors to influence the decision to rechallenge it later.

The overall incidence of any grade of pneumonitis is around 2.7%, and only 0.8% for grade 3–4 pneumonitis [15]. The median time to onset of symptoms varies between 2.3 and 2.8 months, ranging from a minimum of 9 days to as long as 27.4 months [7]. Our patient developed ICI-ILD after 15 months of exposure. In a patient on ICIs presenting with respiratory symptoms, the diagnosis of ICI-related pneumonitis can only be made after ruling out active infections including bacterial, viral, fungal and tubercular disease, and tumour progression [6]. This does not require a bronchoscopy with BAL or TBLB, as long as an HRCT done is classical for ICI-ILD and rules out the other differentials [8, 15, 16]. Our patient had no features suggestive of infection like fever, sputum production and an elevated white blood cell count. Since coronavirus-19 (COVID-19) was prevalent too, COVID-19 RT-PCR, RAT as well as CBNAAT were done, all of which were negative. In our patient, the HRCT chest showed a classical NSIP pattern of ILD, which is the second most common type of ICI-ILD radiologically (after cryptogenic organising pneumonia) [15].

Most of the cases of pneumonitis are mild, where management includes primarily temporary discontinuation of ICI [15]. For grade 3 or 4 pneumonitis, ESMO and ASCO guidelines [8, 16] recommend high dose intravenous steroids, and additional immunosuppression with MMF in patients with no response, as well as permanent discontinuation of the ICIs. This is not based on much evidence, and re-introduction might be tried in cases where there are no options left. However, it is known that patients with a severe primary episode (grade 3 or more), relapse will occur prematurely [7], and that recurrent pneumonitis is usually more severe than the first episode [17].

In our patient, in view of progression on single-agent pazopanib, it was imperative to start some therapy as soon as possible. We had two options, dacarbazine and restarting pembrolizumab. Since dacarbazine is associated with dismal outcomes, and our patient had a remarkable response to pembrolizumab, re-challenging pembrolizumab was the preferred option. Various prophylactic strategies have been suggested for the rechallenge, including concurrent immunosuppression. Initial evidence came from studies on ICI-related gastrointestinal toxicity, where the addition of vedolizumab or infliximab was associated with a significantly lower risk of recurrent adverse events [18, 19]. Another case series of patients with immune-mediated arthritis reported successful re-initiation of iplimumab with concurrent tocilizumab [20].

Recent data has shown that IL-6 is an important cytokine associated with the development of irAEs Additionally, blocking both IL-6 and PD-1/PD-L1 provides synergistic anti-tumour effect [10]. IL-6 is a pleiotropic cytokine that induces immune tolerance to tumour antigens [21, 22]. It has been identified as a predictive [23] and prognostic marker [24] of ICI response as well. Blockade of the IL-6 signalling can enhance anti-tumour immunity and help in better control of disease, apart from prevention of irAE. Anti-IL6 therapy has been hypothesised to be effective in patients with ICI-pneumonitis [6]. Tocilizumab has been used previously as a prophylactic strategy for the prevention of irAEs, as well as a strategy for steroid refractory cases [4, 25]. It was associated with clinical improvement in 79.4% of patients when given concurrently with nivolumab in patients with lung cancer.

Based on this data, it seemed plausible that blocking the IL-6 pathway would help reduce the risk of recurrent pneumonitis with ICIs. Hence, we re-introduced pembrolizumab, along with tocilizumab infusion with every dose every three weeks. The patient tolerated this well, and after 3 months of this combination therapy, a response assessment PET scan revealed complete resolution of the new lesions and did not show any worsening pneumonitis. The patient has currently completed 15 cycles and continues to be in sustained CMR with no significant toxicities.

## Conclusion

Immunotherapy has emerged as an effective therapy against multiple cancers, even ones that were once thought to be immune-quiescent, like STSs. Severe grade 3 or 4 immune-related adverse effects can be life-threatening and have till now been an indication for permanent discontinuation of ICIs. Through this case report, we have tried to show how concurrent immunosuppression with anti-IL-6 therapy tocilizumab can help us utilise the benefit of immunotherapy despite the development of high-grade irAEs. This adds to the limited data that has demonstrated the successful use of infliximab, vedolizumab and tocilizumab in patients with colitis and arthritis previously. Our patient tolerated the re-challenge of ICIs with tocilizumab without recurrence of pneumonitis. This merits future prospective trials to include this combination in routine practice.

## Informed consent statement

Informed consent was taken from the patient before writing the case report.

## Conflicts of interest

No author has any relevant financial and/or nonfinancial conflicts of interest.

## Funding

No financial and/or material support or funded writing assistance was received for this work.
